# Supplementary figures and images for: Metabolic recovery and compensatory shell growth of juvenile Pacific geoduck Panopea generosa following short-term exposure to acidified seawater
Source: Conserv Physiol. 2020 Apr 4;8(1):coaa024. doi: 10.1093/conphys/coaa024 (PMC7125045; doi:10.1093/conphys/coaa024)

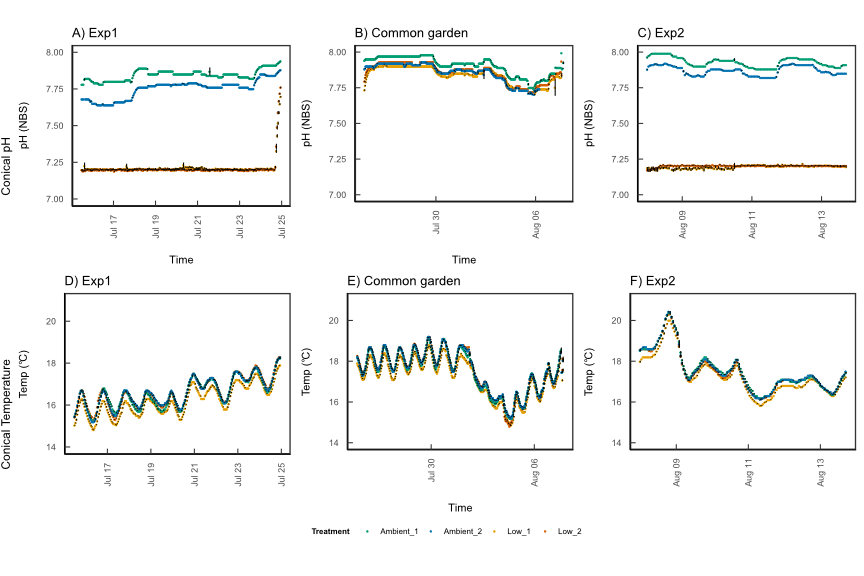

Supplement: Supplementary_Figure_1_coaa024 [file supplementary_figure_1_coaa024.jpeg]
